# Tissue metabolite of type I collagen, C1M, and CRP predicts structural progression of rheumatoid arthritis

**DOI:** 10.1186/s41927-019-0052-0

**Published:** 2019-01-31

**Authors:** Anne C. Bay-Jensen, Adam Platt, Martin A. Jenkins, Michael E. Weinblatt, Inger Byrjalsen, Kishwar Musa, Mark C. Genovese, Morten A. Karsdal

**Affiliations:** 1 Rheumatology, Nordic Bioscience, Biomarkers and Research, Herlev Hovedgade 205-207, 2730 Herlev, Denmark; 2 Precision Medicine and Genomics, IMED Biotech Unit, AstraZeneca, Cambridge, UK; 3 Global Medicines Development, AstraZeneca, Cambridge, UK; 4 Division of Rheumatology, Immunology and Allergy, Brigham and Women’s Hospital, Boston, MA USA; 5 Division of Immunology and Rheumatology, Stanford University, Palo Alto, California, USA

**Keywords:** Rheumatoid arthritis (RA), Biomarkers, Radiographic progression, And trial enrichment

## Abstract

**Background:**

Biomarkers of rheumatoid arthritis (RA) disease activity typically measure inflammation or autoimmunity (e.g. CRP, RF). C1M and C3M, metabolites of type I and III collagen, are markers reflecting tissue metabolism. These markers have been documented to provide additional prognostic and predictive value compared to commonly used biomarkers. We investigated the relationship of high serum levels of C1M or C3M to radiographic progression, and benchmarked them to CRP and RF.

**Methods:**

Placebo treated patients of the OSK1, 2 and 3 studies (Phase III clinical trials testing efficacy of fostamatinib) with baseline serum biomarkers C1M, C3M, CRP and RF were included (n_BL_ = 474). Van der Heijde mTSS was calculated at baseline and 24-week (n_24_ = 261). Progression was defined as moderate or rapid by ΔmTSS ≥0.5 or ≥ 5 units/year. Patients were divided into subgroups; low (L), high (H) or very high (V) C1M, C3M and CRP, or RF negative, positive and high positive. Difference in clinical parameters were analyzed by Mann-Whitney or χ^2^tests, and modelling for prediction of progression by logistic regression including covariates (age, gender, BMI, and clinical assessment scores).

**Results:**

Levels of C1M, C3M, CRP and RF were significantly (*p* < 0.05) associated with measures of disease activity and mTSS at baseline. For prognostic measures, there were 2.5 and 4-fold as many rapid progressors in the C1M_H_ and CRP_H_ (p < 0.05), and in the C1M_V_ and CRP_V_ groups (*p* < 0.001) compared C1M_L_ and CRP_L_, respectively. C1M and CRP performed similarly in the predictive analysis, where high levels predicted moderate and rapid progression with odds ratio of 2.1 to 3.8 and 3.7 to 13.1 after adjustment for covariates. C3M and RF did not provide prognostic value alone.

**Discussion:**

Serum C1M and CRP showed prognostic value and may be tools for enrichment of clinical trials with structural progressor. The two markers reflect two different aspect of disease pathogenesis (tissue turnover vs. inflammation), thus may provide individual and supplementary information.

**Electronic supplementary material:**

The online version of this article (10.1186/s41927-019-0052-0) contains supplementary material, which is available to authorized users.

## Introduction

Rheumatoid arthritis (RA) is a chronic autoimmune disease characterized by poly-articular inflammation of synovial joint tissue resulting in irreversible joint destruction and bone erosions [1]. During the course of disease, the speed of joint destruction (progression) is heterogeneous amongst RA patients, thus measurement of disease activity (e.g. disease activity score (DAS)) or joint damage (e.g. modified total sharp score (mTSS)) at a given time point is not necessarily predictive of the rate of progression. It is challenging to identify which patients will progress, which may be those that are in most need of treatment and have the greatest potential for response to treatment [2]. In intervention trials, the number of patients required to demonstrate structural efficacy is tightly linked to the proportion of the enrolled patients progressing during the trial. The rate of progression is the basis for the sample size calculation, i.e. the required sample size can be decreased either by following patients for an extended period of time or by enrolling patients with a higher probability of disease progression. Thus, smaller and more effective clinical trials could be designed if prognostic biomarkers (e.g. biochemical) could be used to enrich study populations for disease progressors.

Type I and type III collagen are amongst the most abundant proteins in the body and the joint [3] and they are known to be modulated by inflammation. Bone consists of largely type I collagen, which is resorbed by mainly Cathepsin K in normal homeostasis while in RA, there is a shift in the balance towards a matrix metalloproteinase (MMP) driven degradation observed as erosions [4]. A strong association between erosion and bone turnover and C1M released to the circulation is therefore probable. Making C1M a direct measure of inflammation driven bone and soft tissue turnover.

Tissue destruction and bone erosion are driven by accelerated proteolytic activity induced by inflammation [5, 6]. Both preclinical and clinical studies have shown that turnover of type I and type III collagen is significantly increased in RA, shifting the equilibrium toward a net degradation of collagen [7, 8]. The degradation of tissue collagens is mediated by enzymatic cleavage predominantly by MMPs. MMPs, such as MMP3, have been shown to be highly upregulated in RA [9]. The action of MMPs on collagen results in the release of protein fragments [8], collagen metabolites, into the circulation. Numerous studies have shown that measurement of these metabolites may serve as disease activity markers or predictive markers of progression [10]. The type I and type III collagen metabolites C1M and C3M were both shown to be correlated to disease activity and burden of disease, however only C1M was associated with progression [11–13]. Other markers have shown predictive capabilities. Aletaha et al. demonstrated that rheumatoid factor (RF) is predictive of progression [14]. Likewise studies dating back to early 2000s have shown similar potential of C-reactive protein (CRP) [15].

This may be the first study that investigate and compare the predictive value of tissue related markers and inflammatory markers in a patient population with moderate to severe RA. The aim of the present study was to investigate whether high levels of the type I and type III collagen metabolites C1M and C3M at baseline, as well as RF and CRP, could enrich for progressors in the placebo group of the phase III OSKIRA 1, 2 and 3 clinical trials [16].

## Patients and methods

### Data source

Biomarkers were measured in all patients of the placebo arm who consented and where serum samples were available at baseline in the OSKIRA-1 study (NCT01197521) [17] according to a prospective biomarker analysis plan. Additionally, the placebo arms of the OSKIRA-2 (NCT01197534) and − 3 studies (NCT01197755) were assessed according to a retrospective biomarker plan. All placebo patients received methotrexate (MTX) and received active therapy (100 mg fostamatinib BID) from week 12 if not responding at that time point. Brief summaries of the individual studies are provided in the supplementary data (Additional file 1: Study descriptions).

**Fig. 1:**
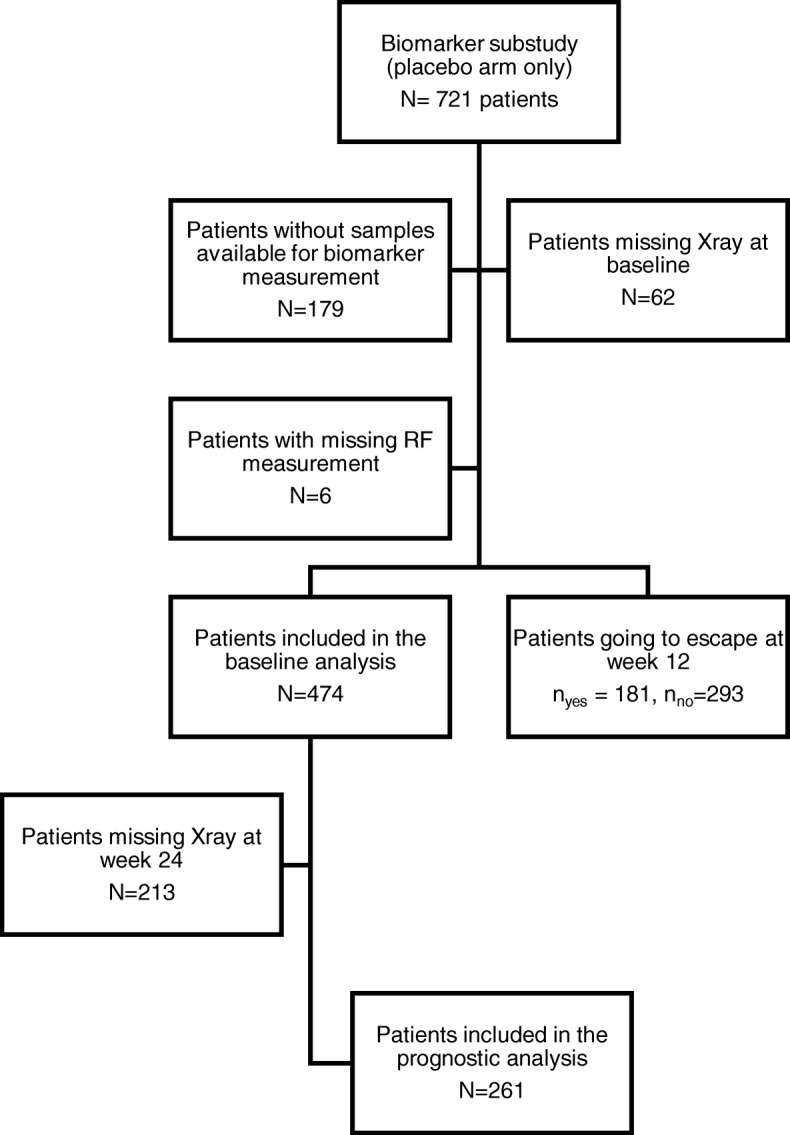
Flow of current biomarkers study design

Data from the three trials were pooled and used as a single data set. Radiographic data using van der Heijde modified total Sharp scores (mTSS) were available from all trials. Radiographic changes were calculated by subtracting the baseline mTSS score from the score at 24 months, and radiographic progression was defined as changes ≥0.23 mTSS units linear extrapolated from ≥0.5 units/year [18]. In addition, we defined rapid radiographic progression as an increase of ≥5 units/year. These cut-offs have previously been used in similar analysis [14, 18, 19]. Following measures was recorded for all included patients: swollen joint counts and tender joint counts (SJC, TJC) on a 28-joint scale, the patient global assessment and evaluator global assessment of disease activity (PGA, EGA, based on a visual analogue scale from 0 to 10 cm), the C reactive protein (CRP in mg/dL), rheumatoid factor status (RF positive (+) ≥20 U/mL) and erythrocyte sedimentation rate (ESR, in mm/h), Health Assessment Questionnaire (HAQ, 0–3) function and pain scores (10 cm visual analogue scale). Assessment of anti-CCP was not included in the analysis as data was missing from substantial part of the patients. C1M (nmol/L) and C3M (nmol/L) were measured in serum by quantitative competitive enzyme linked-immunosorbent assays (ELISAs) [11, 12, 20]. All samples were: measurable (>lower quantification level), run in duplicates and rerun if above CV% was above 15%. Three internal and two kit controls were included on each plate. Inter- and intra-assay CV < 15%. C1M and C3M levels for each of the three studies can be found in supplementary data (Additional file 1 : Table S1). A study flow-diagram the subgroup analysis can be seen in Fig. 1.

### Statistical analysis

Patients with baseline biomarkers available were sub-grouped according to biomarker levels or RF status. Levels of C1M, C3M or CRP above or below the median were considered as low (L) and high (H) levels, whereas the highest quartile levels (top 25%) were considered as very high (V) levels. Besides comparing the RF positive (H) and negative (L) subgroups, we also defined a subgroup of patients as RF high positive (V), which were patients with above the median in the positive group [14]. We investigated differences in baseline disease activity, severity and radiographic progression between the different subgroups. This was done by Mann Whitney test and chi-squared as appropriate. We multivariate logistic regression to test whether baseline level C1M, C3M, CRP and RF were predictive of progression, adjusting for baseline covariates, such as age, gender, health and disease activity assessments, as well as radiographic scores. Testing of the additive predictive value of the biomarkers when combined were likewise tested by logistic regression adjusting for age, gender, health and disease activity assessments.

## Results

### Patients

A pooled dataset of 474 placebo treated patients (all on MTX) was generated by retrieving patient data from the three OSKIRA 1, 2 and 3 studies: From 721 patients in total, 185 were excluded as blood samples or RF data were missing at baseline at time of the explorative biomarker study (Fig. 1). In addition, 62 patients were excluded due to missing baseline radiography scoring. 261 patients had radiographic scores available at week 24 and could be used for predictive analyses of progressors. Of the 474 patients, 184 went into escape therapy at week 12 (Fig. 1), due to lack of response to MTX.

The studies were balanced on gender, age and BMI (Table 1). There were relatively more Caucasians and fewer Hispanic/Latinos in OSKIRA 2 compared to the other studies. Disease duration, but not DAS28, was significantly different between the studies. Background treatment at baseline and previous use of anti-TNF was different between the studies as per protocol.

**Table 1:** Patient characteristics in each of the phase III clinical studies, OSKIRA-1, -2 and -3.

Study		OSKIRA 1	OSKIRA 2	OSKIRA 3	p
N		206	197	71	
Gender, n (%)	Females	173 (84%)	169 (86)	55 (78)	> 0.1
Age		53.0 (12.0)	54.4 (12.5)	54.1 (12.7)	> 0.1
BMI		28.0 (6.1)	28.0 (6.4)	29.7 (6.7)	> 0.1
Ethnicity, n (%)	African decent	4 (2)	13 (6.6)	6 (8.4)	< 0.0001
	ASIAN	1 (0.5)	20 (10.2)	0	
	Caucasian	108 (52.4)	150 (76.1)	37 (52.1)	
	Hispanic or Latino	93 (45.1)	12 (6.1)	28 (39.4)	
	Other	0	2 (1.0%)	0	
Disease duration, years (SD)		9.1 (7.9)	7.5 (8.6)	10.2 (7.8)	< 0.0001
Disease activity score, mean (SD)		5.9 (0.8)	5.6 (0.9)	5.6 (0.4)	> 0.1
Presence of erosions at baseline	Yes	100 (49.8)	93 (47.2)	37 (52.1)	> 0.1
Previous conventional DMARDs	Yes	76 (36.9)	64 (32.5)	22 (31.0)	> 0.1
Previous anti-TNFa treatment	Yes	10 (4.9%)	36 (18.3%)	71 (100%)	< 0.0001
Previous other biologics	Yes	0 (0%)	0 (0%)	0 (0%)	> 0.1
Escape at week 12	Yes	66 (32.0)	90 (45.7)	25 (35.2)	0.016

Data is presented as mean (SD) or frequency (%) as appropriated. Significant difference between studies was estimated by either χ^2^ or Kruskal-Wallis test (alpha = 0.05)

### Association between biomarker subgroups and disease activity or severity

Baseline levels of disease activity and clinical assessment scores, for each of the biomarker sub-groups are shown in Table 2. There was no difference in mean age in the biomarker groups. There were significantly more females in the C1M low and CRP low groups compared to the high and very high groups. There was a strong association between the C1M, C3M and CRP, whereas there were no association between C1M and RF. Patients with high and very high levels of C1M, C3M and CRP had significantly higher DAS28 score (*p* < 0.0001). The three markers were associated with SJC (*p* < 0.01), however less so to TJC. High CRP as well as very high C1M and CRP, were strongly associated with patient reported scores, whereas C3M was less so and RF was not associated. High and very high levels of RF and CRP were strongly associated with the radiographic scores (*p* < 0.001). High and very high levels of C1M and C3M were likewise associated with radiographic scores though less significantly (*p* < 0.05).

**Table 2:** Summary measures and comparison of baseline patient characteristics between the different C1M, C3M and RF subgroups

	All	C1M_L_	C1M_H_	C1M_V_	C3M_L_	C3M_H_	C3M_V_	RF_L_	RF_H_	RF_V_	CRP_L_	CRP_H_	CRP_V_
N	474	237	237	118	237	237	118	94	380	198	240	234	118
Gender (%)		87.8	79.7*	74.6**	84.0	83.5	83.1	84.0	83.7	84.0	87.1	80.3*	77.1*
Age, years		53.8 (12.1)	53.7 (12.6)	54.1 (13.0)	53.8 (12.2)	53.4 (12.5)	53.2 (11.2)	54.9 (12.7)	53.4 (12.2)	54.4 (11.5)	53.7 (12.2)	53.8 (12.5)	54.0 (12.2)
BMI, kg/cm^2^	28.2 (6.3)	27.9 (6.2)	28.6 (6.4)	28.8 (6.6)	27.9 (6.1)	28.6 (6.4)	28.9 (6.0)*	28.7 (7.1)	28.1 (6.1)	28.2 (5.8)	27.9 (6.1)	28.5 (6.5)	28.6 (6.8)
Disease duration, years	8.6 (8.2)	8.9 (8.8)	8.4 (7.7)*	8.0 (7.3)	8.1 (8.5)	9.2 (7.9)*	9.2 (8.0)*	7.2 (8.9)	9.0 (8.0)**	9.7 (7.4)****	8.3 (8.3)	9.0 (8.1)	8.7 (7.4)
C1M, nmol/mL	103 (80)	47 (16)	159 (779)****	216 (76)****	72 (49)	134 (92)****	157 (103)****	96 (84)	105 (79)	106 (79)	56 (25)	150 (88)****	193 (94)****
C3M, nmol/mL	34.1 (15)	29.7 (12.5)	38.4 (15.9)****	42.4 (15.2)****	23.6 (4.6)	44.6 (14.3)****	53.7 (15.6)****	27.7 (10.7)	35.6 (15.4)***	40.9 (17.8)****	28.3 (10.3)	39.9 (16.7)****	44.4 (18.4)****
RF, Units/mL	230 (400)	226 (370)	233 (428)****	263 (497)	117 (174)	342 (515)****	489 (651)****	10 (4)	284 (430)****	494 (514)****	153 (228)	308 (509)****	356 (579)****
CRP, mg/dL	15.6 (21.1)	6.1 (8.8)	25.0 (25.2)****	39.0 (29.0)****	8.5 (10.6)	22.6 (26.1)****	30.9 (31.0)****	12.2 (19.2)	16.4 (21.5)**	18.7 (22.3)****	3.8 (2.3)	27.9 (24.7)****	42.9 (27.2)****
DAS	5.7 (0.9)	5.4 (0.8)	6.1 (0.8)****	6.4 (0.8)****	5.5 (0.8)	6.0 (0.9)****	6.2 (0.9)****	5.6 (0.8)	5.8 (0.9)*	5.9 (1.0)**	5.3 (0.7)	6.2 (0.8)****	6.5 (0.8)****
SJC28	12.0 (5.5)	11.7 (5.5)	12.3 (5.6)**	13.2 (5.9)*	11.1 (5.0)	12.9 (5.9)**	13.2 (5.9)**	11.0 (5.3)	12.2 (5.6)*	12.7 (6.0)	11.2 (5.2)	12.9 (5.8)**	13.1 (5.8)**
TJC28	15.7 (6.2)	15.2 (6.0)	16.1 (6.3)	16.8 (6.4)*	15.1 (5.6)	16.2 (6.6)	16.8 (6.8)*	15.1 (6.1)	15.8 (6.2)	16.2 (6.6)	15.0 (5.8)	16.3 (6.5)*	16.7 (6.5)*
HAQ	1.6 (0.6)	1.5 (0.6)	1.6 (0.6)**	1.8 (0.6)****	1.5 (0.6)	1.6 (0.6)**	1.8 (0.6)***	1.4 (0.6)	1.6 (0.6)***	1.7 (0.6)***	1.4 (5.8)	1.7 (0.6)****	1.8 (0.6)****
HAQ PAIN	61.2 (19.2)	57.9 (18.0)	64.5 (19.9)*	69.8 (18.0)****	59.5 (18.1)	62.9 (20.1)*	64.7 (19.7)**	60.1 (19.4)	61.5 (19.2)	62.4 (19.6)	56.5 (18.3)	66.1 (19.0)****	69.7 (18.6)****
Evaluators global assessment	61.7 (16.1)	59.7 (16.2)	63.8 (15.8)*	67.0 (15.0)***	59.9 (16.2)	63.6 (15.8)*	64.9 (14.8)*	59.7 (16.6)	62.2 (16.0)	63.9 (14.7)	59.3 (16.2)	64.2 (15.7)**	66.4 (14.8)***
Patients global assessment	60.2 (19.7)	57.1 (18.8)	63.3 (20.1)*	69.2 (17.8)****	58.3 (18.6)	62.0 (20.6)*	64.6 (19.4)**	60.1 (18.9)	60.2 (19.9)	60.2 (21.1)	55.8 (19.1)	64.7 (19.3)****	68.4 (19.1)****
MTSS	46.8 (63.2)	42.1 (61.2)	51.4 (64.9)*	58.0 (68.6)**	40.8 (59.3)	52.7 (66.4)*	66.8 (75.6)***	25.2 (48.7)	52.1 (65.2)****	62.2 (71.6)****	35.2 (53.4)	58.6 (70.0)***	66.3 (69.9)****
JSN	24.0 (30.9)	21.5 (30.0)	26.5 (31.7)*	29.1 (33.0)**	21.0 (29.5)	27.0 (32.0)*	33.7 (35.9)***	12.9 (23.9)	26.7 (31.8)****	31.8 (34.4)****	18.1 (23.3)	30.2 (33.9)****	33.7 (32.9)****
ERN	22.8 (33.7)	20.6 (32.8)	24.9 (34.5)	28.9 (37.3)*	19.8 (31.0)	25.7 (36.0)	33.1 (41.3)**	12.3 (25.4)	25.3 (35.0)***	30.4 (39.2)****	17.2 (28.4)	28.4 (37.6)****	32.6 (38.6)****

Data is shown as mean (SD) unless otherwise indicated. Mann-Whitney was used to compare the highest subgroups with the low/− (alpha = 0.05), significance levels are indicated by * (p ≤ 0.05), ** (p < 0.01), *** (p < 0.001) and **** (p < 0.0001)

### Biomarker subgroups and radiographic progression

There were significantly more radiographic progressors in the high and very high C1M subgroups (37 and 43%, Table 3) compared to the low C1M subgroup (26%, *p* = 0.045) corresponding to an enrichment of progressors of 42 and 65%, respectively. There were also significantly more rapid progressors in the high and very high C1M group as compared to the low group (14 and 21% vs. 6%, *p* < 0.05, Additional file 1: Table S3) corresponding to a 2.3- and 3.5-fold enrichment of progressors, respectively. There were significantly more radiographic progressors in the very high CRP subgroups (46%, Table 3) compared to the low CRP subgroup (27%, *p* = 0.013) corresponding for an enrichment of 70%. There were also significantly more rapid progressors in the high and very high CRP group as compared to the low group (14 and 24% vs. 6%, p < 0.05, Table 3) corresponding to a 2.3- and 4-fold enrichment of progressors, respectively. The overlap between patients identified by C1M and CRP was 80%; 24 patients were uniquely identified by C1M. The frequency of progressors was between 37 and 46% independent of the level of CRP, when C1M was high or very high. Whereas when C1M was low at any level of CRP only up to 29% progressor was found. The group of patients with both high C1M and CRP included 16% rapid progressors (3% more than either marker alone). Neither high/very high C3M nor RF+/high RF+ subgroups contained significantly more progressors; however, there was a trend in the high RF+ subgroup of having more progressors and rapid progressors than the RF subgroup (Table 3).

**Table 3:** Disease progression from baseline to week 24

	N	% progressors (delta mTSS > = 0.5 units per year)		% Rapid progressors (delta mTSS > = 5 units per year)		Delta sharp score		Delta erosion Score		Delta Joint space narrowing score	
C1M_L_	145	25.5%		5.5%		0.23 (1.66)		0.01 (0.77)		0.20 (1.19)	
C1M_H_	116	37.1%	0.045	13.8%	0.022	0.56 (3.16)	> 0.1	0.48 (2.49)	0.066	0.08 (1.07)	> 0.1
C1M_V_	51	43.1%	0.019	21.6%	0.0009	1.19 (4.31)	0.031	0.99 (3.47)	0.025	0.21 (1.28)	> 0.1
C3M_L_	133	31.6%		7.5%		0.43 (2.60)		0.22 (1.85)		0.20 (1.24)	
C3M_H_	128	29.7%	> 0.1	10.9%	> 0.1	0.33 (2.59)	> 0.1	0.23 (1.69)	> 0.1	0.10 (1.02)	> 0.1
C3M_V_	59	28.8%	> 0.1	13.6%	> 0.1	0.33 (2.29)	> 0.1	0.36 (2.12)	> 0.1	−0.03 (1.09)	> 0.1
CRP_L_	145	26.9%		5.5%		0.18 (1.48)		−0.00 (0.78)		0.16 (1.02)	
CRP_H_	116	35.3%	> 0.1	13.8%	0.022	0.63 (3.26)	> 0.1	0.51 (2.49)	0.058	0.13 (1.27)	> 0.1
CRP_V_	50	46.0%	0.013	24.2%	0.0006	1.40 (4.52)	0.025	1.06 (3.50)	0.0074	0.34 (1.70)	> 0.1
RF_L_	53	24.5%		3.8%		0.00 (0.95)		−0.06 (0.52)		0.06 (0.62)	
RF_H_	208	32.2%	> 0.1	10.6%	> 0.1	0.48 (2.69)	> 0.1	0.29 (1.96)	> 0.1	0.17 (1.23)	> 0.1
RF_V_	104	34.6%	> 0.1	13.5%	0.059	0.44 (2.64)	> 0.1	0.20 (1.84)	> 0.1	0.22 (1.36)	> 0.1

Data is shown as mean (SD) unless otherwise indicated. Chi-squared test or Mann-Whitney test was used as appropriate to compare C1M, C3M, CRP and RF subgroups (alpha = 0.1) and significance levels are indicated by exact *p* values

Progression of joint damage assessed by Delta mTSS score was lower in the low C1M and CRP groups (0.23 and 0.18, respectively) compared to the very high group (1.19 and 1.40, *p* < 0.05, Table 3). There were no differences between either the C3M or RF subgroups in radiographic changes over the 24-week period.

### Prognostic value of C1M and CRP

Next, we investigated the association of disease activity measures in the high and very high C1M and CRP with prediction of radiographic progression as measured by mTSS. All significant demographic and clinical variables from Table 2 were included as covariables. High levels of C1M could significantly (p < 0.05) predict progression and rapid progression with odds ratios (ORs) of 2.1 and 3.7 (Table 4). Very high levels of C1M could likewise significantly (*p* < 0.01) predict with progression and rapid progression with ORs of 1.3 and 1.7. Similar results were observed for CRP; high levels could predict progression and rapid progression with ORs of 2.1 and 4.1 (*p* < 0.05), whereas very high levels could predict with ORs of 1.3 and 1.7 (p < 0.01). The combination of the two markers provided a markedly higher OR for progression (Table 4). The best prognosis was from selecting patients with high levels of C1M and very high levels of CRP giving a odds ratio of 3.8 and 13.1 (*p* < 0.001). In this case, less than 20% of the population would be selected.

**Table 4:** Prediction of progression and rapid progression by logistic regression. The odds ratio (ORs) were adjusted for the variables in Table 2

	n (%)	Progression			Rapid progression		
		OR	95% CI	P	OR	95% CI	P
C1M_H_	116 (44.4)	2.05	1.13 to 3.71	0.018	3.74	1.36 to 10.3	0.011
C1M_V_	51 (19.5)	1.29	1.07 to 1.55	0.0070	1.67	1.27 to 2.19	0.0003
CRP_H_	116 (44.4)	2.08	1.12 to 3.84	0.020	4.13	1.48 to 11.5	0.0067
CRP_V_	50 (19.2)	1.33	1.11 to 1.60	0.0021	1.73	1.31 to 2.27	0.0001
C1M_H_ + CRP_H_	92 (35.2)	2.51	1.27 to 4.98	0.0085	5.87	1.85 to 18.6	0.0026
C1M_H_ + CRP_V_	46 (17.6)	3.82	1.89 to 8.51	0.0011	13.1	3.6 to 48.0	0.0001
C1M_V_ + CRP_H_	51 (19.5)	3.14	1.44 to 6.86	0.0046	9.43	2.83 to 31.4	0.0003
C1M_V_ + CRP_V_	36 (13.8)	3.64	1.57 to 8.44	0.0026	11.5	3.3 to 40.7	0.0001

### Biomarker levels for escape and non-escape patients

Of the 474 patients included for the baseline subgrouping, 181 patients went to escape therapy at week 12 and therefore not part of the radiographic follow-up analyses. Thus, we investigated the level of the biomarkers at baseline in escape and non-escape patients. The baseline levels of C1M and CRP were significantly higher in escape patients compared to the non-escape (Table 5). Furthermore, the frequencies of C1M high and CRP high and very high patients were significantly higher (p < 0.05) in the escape group compared to the non-escape.

**Table 5:** Biomarker levels at baseline in escape and non-escape patients. Patients with inadequate response to the therapy went to escape at week 12. The levels and proportions were compared by Mann-Whitney test^a^ or Chi-squared test^b^

Variable	Escape patients			Non-escape patients				
	Mean (SD)	n	No biomarker high and very high patients (%)	Mean (SD)	P^a^	n	No biomarker high and very high patients (%)	P^b^
C1M	119 (93)	181	102 (56%) 57 (32%)	93 (69)	0.0019	293	135 (46%) 61 (21%)	0.030 0.0091
C3M	35.2 (15.3)	181	93 (51%) 52 (29%)	33.4 (14.7)	> 0.1	293	144 (49%) 66 (23%)	> 0.1 > 0.1
RF	233 (362)	181	141 (78%) 79 (44%)	227 (422)	> 0.1	293	239 (82%) 118 (40%)	> 0.1 > 0.1
CRP	20.4 (26.2)	181	102 (56%) 61 (34%)	12.6 (16.6)	0.0009	293	132 (45%) 57 (20%)	0.017 0.0001

## Discussion

Both C1M and C3M are metabolites of type I and III collagen, the most abundant joint tissue collagens, released due to an up-regulation of MMP activity as a result of either flare or continued inflammation in the connective tissues [7]. CRP is an acute phase reactant and RF a measure of immunoglobulins. The biomarkers reflect different pathological processes driven by the underlining chronic inflammation. C1M and C3M have previously been applied as pharmacodynamic markers [11, 20, 21], whereas RF has typically been used to classify patients as an inclusion criteria for clinical trials [22]. CRP has predominantly been used as both a diagnostic and a pharmacodynamic measure, going back to the early 1970s [23, 24]. C1M and C3M are newer markers; C1M has previously been shown to be predictive of structural progression in RA [12] and C3M has been shown to be associated with disease activity scores [11, 13]. Moreover, both C1M and C3M have been shown to reflect response to anti-inflammatory treatment [11, 20, 21]. As Aletaha et al. [14] demonstrated, RF was partly predictive of structural progression in RA. We performed similar analyses to confirm and build upon their data. We pooled the available biomarker data from the placebo arms from the three OSKIRA-1, − 2 and − 3 trials and demonstrated that all four biomarkers are significantly associated with measures of disease activity and severity.

While all four serum biomarkers were associated with disease activity as measured by DAS28 and HAQ pain at baseline, there was no association between RF and C1M, which may give some insight into the differences between tissue derived biomarkers and direct measures of inflammation and autoimmunity. C3M was significantly higher in the RF positive sub-groups, indicating that RF positive patients tend to have higher levels of C3M. C3M, however is not a prerequisite for high RF. All four markers were associated with structural severity at baseline, however only C1M and CRP could significantly identify more structural progressors by delta change in mTSS. High C1M and high CRP were alone predictive of progression with OR up to 4. We saw very similar patterns for C1M and CRP, which could indicate that the markers would perform equally well in enrichment for patients with at structural progressive disease. However, we did observe an improvement of the OR by combining C1M and CRP reaching ORs up to 13. The two markers are distinct in their molecular origin. CRP is released mainly from the liver and act as an acute reactant, whereas C1M is released from the inflamed tissue and therefore a direct measure of tissue turnover [25]. The strong dependency of the markers may be explained by the common connection to inflammation and that CRP is an upstream modulator of tissue turnover. Hints to the difference between the two markers can be found in aforementioned phase III clinical study LITHE, testing the efficacy of tocilizumab. Tocilizumab completely and instantly suppressed the level of CRP. In contrast, C1M is gradually suppressed over time in response to treatment underlining that the two markers are somewhat differentially modulated [26]. An interesting observation is that C1M is elevated and associated with synovitis in osteoarthritis, which is normally not considered as an inflammatory disease [27]. Although C1M and CRP are equally predictive of progression in this study, they provide independent information.

These data indicate that disease activity, structural disease burden and progression are associated with different molecular processes; radiographic progression seems to be linked to elevated type I collagen turnover and CRP levels. This is consistent with previous data demonstrating that C3M was associated with disease activity and current state of disease [11, 28] while type I collagen turnover measured by C1M may be connected to an ongoing process of joint deterioration [12]. With this finding it is tempting to speculate that C1M measurement may be used as a drug development tool to enrich clinical studies for progressors thereby expanding the therapeutic window: That is increasing the proportion of patients that are more likely to respond to a treatment believed to have joint protective effect, and thereby enhancing the likelihood for reaching clinical significance. We performed power calculations which indicate that studies designed similarly to OSKIRA studies can be enriched for progressors using C1M, and thereby reduce the patient numbers needed by 50% (Additional file 1; Table S2). This number could have massive effect on the economic burden of conducting a clinical study and potentially impact how clinical studies are designed in the future, with greater emphasis on structural protection afforded through a novel drug candidate. Furthermore, C1M might be used in clinical practice to move a subset of patients to medicine with a different mode of action if they still have high C1M post treatment. Both applications require further clinical validation and qualification. CRP showed similar pattern as C1M and could be applied in similar manner. The major difference is that CRP is acute phase reactant and C1M is a tissue turnover measure thus they may complement each other, certify an enrichment decision.

A limitation of the study is that the prognostic biomarker analyses were derived using data from patients who had both baseline and week 24 biomarker data available, not on the complete set of patients who had consented to provide samples for biomarker analysis. Thus, escape patients are missing from the prognostic analyses, which may result in the exclusion of patient data with the highest level of the biomarkers and therefore potentially the most active and progressive disease. Furthermore, patients were included into the study partly based on their CRP levels. This means that only patient above a given threshold of CRP thus biased toward very high levels of CRP. Additionally, since the assessment of biomarkers in the OSKIRA studies was exploratory endpoints, the clinical study was not sized for the analyses of biomarker data and their anticipated treatment effect. Lastly, the patients were all inadequate responders to either MTX, DMARDs or anti-TNF, which are very common phase III populations, however may provide shewed progression ratio as compared to overall RA population.

## Conclusions

Biomarkers have commonly been used in clinical trials as mainly explorative endpoints or pharmacodynamic markers. However, we propose a role for biomarkers in patient selection and clinical trial enrichment to support drug development and improve success rates. We found that C3M and RF were associated with disease activity and burden, whereas C1M and CRP reflect radiographic progression. These data indicate that C1M, alongside with CRP, could be developed and qualified as a drug development tool for the enrichment of progressors in clinical trials of RA.

## Additional file

Additional file 1: Study descriptions. (DOCX 30 kb)
