# Supplementary material for: Tissue metabolite of type I collagen, C1M, and CRP predicts structural progression of rheumatoid arthritis
Source: BMC Rheumatol. 2019 Jan 31;3:3. doi: 10.1186/s41927-019-0052-0 (PMC6390574; doi:10.1186/s41927-019-0052-0)
Supplement: Supplementary file 1 — Study descriptions. (DOCX 30 kb) [file 41927_2019_52_MOESM1_ESM.docx]

# Additional data

## Study descriptions

Information abstracted from the trial protocols.

### OSKIRA-1

**Title**: A phase III, multi-centre, randomised, double-blind, placebo-controlled, parallel group study of two dosing regiments of fostamatinib disodium in rheumatoid arthritis (RA) patients with an inadequate response to methotrexate.

**Sponsor:** AstraZeneca AB, Sweden

**PI;** Michael E Weinblatt, MD, Brigham and Women’s Hosptial, Boston (MA), USA

**Objective:** The primary objective of this study was to evaluate the efficacy of two oral dosing regiments of fostamatinib in combination with methotrexate, in patients with active RA.

**Patient (in brief):** Patients with active disease defined as at least 6 swollen joints and either ESR of ≥28 mm/hr or CRP ≥10mg/L. Patient should have at least one of following; history of RF positive, RF positive, radiographic erosion within a year of enrolment or presence of anti-CCP. Treatment with oral, subcutaneous or intramuscular methotrexate for at least 6 months prior to randomization.

**Biomarkers:** Exploratory endpoint to investigate systemic profiles in RA patients. Serum and plasma samples were collected for optional exploratory biomarker analysis.

### OSKIRA-2

**Title**: A phase III, multi-centre, randomised, double-blind, placebo-controlled, parallel group study of two dosing regiments of fostamatinib disodium in rheumatoid arthritis (RA) patients with an inadequate response to DMARDs.

**Sponsor:** AstraZeneca AB, Sweden

**PI;** Peter Dawes, MD, University Hospital of North Staffordshire NHS Trust, Stoke-on-Trent, UK

**Objective:** The primary objective of this study was to evaluate the efficacy of two oral dosing regiments of fostamatinib in combination with methotrexate, in patients with active RA.

**Patient (in brief):** Patients with active disease defined as at least 4 swollen or tender joints, and either ESR of ≥28 mm/hr or CRP ≥10mg/L. Patient should have at least one of following; history of RF positive, RF positive, radiographic erosion within a year of enrolment or presence of anti-CCP. Treatment with 1 of following traditional DMARDs: methotrexate, sulfasalazine, hydroxychloroquine or chloroquine for at least 6 months.

**Biomarkers:** Exploratory endpoint to investigate systemic profiles in RA patients. Serum and plasma samples were collected for optional exploratory biomarker analysis.

### OSKIRA 3

**Title**: A phase III, multi-centre, randomised, double-blind, placebo-controlled, parallel group study of two dosing regiments of fostamatinib disodium in rheumatoid arthritis (RA) patients with an inadequate response to TNF-alpha antagonist.

**Sponsor:** AstraZeneca AB, Sweden

**PI;** Mark Genoves, MD, Stanford University Medical center, Palo Alto (CA), USA

**Objective:** The primary objective of this study was to evaluate the efficacy of two oral dosing regiments of fostamatinib in combination with methotrexate, in patients with active RA.

**Patient (in brief):** Patients with active disease defined as at least 6 swollen or tender joints, and either ESR of ≥28 mm/hr or CRP ≥10mg/L. Patient should have at least one of following; history of RF positive, RF positive, radiographic erosion within a year of enrolment or presence of anti-CCP. Treatment with oral, subcutaneous or intramuscular methotrexate for at least 6 months prior to randomization. Received treatment with a single TNF-alpha antagonist for 12 weeks up to randomization, unless discontinued for tolerability and safety reasons, and designated as a treatment failure due to lack of efficacy, safety, or tolerability.

**Biomarkers:** Exploratory endpoint to investigate systemic profiles in RA patients. Serum and plasma samples were collected for optional exploratory biomarker analysis.

## Table S1. Marker levels in the different studies.

|  | Low levels | High levels | Very high levels | P-value |
| --- | --- | --- | --- | --- |
| C1M | 21.2 – 67.2 | 67.3 – 396 | 123 - 396 |  |
| All studies | 50% | 50% | 25% |  |
| OSKIRA 1 (125) | 66 (52.8%) | 59 (47.2%) | 28 (22.4%) | 0.5528  0.5577 |
| OSKIRA 2 (93) | 46 (49.5%) | 47 (50.5%) | 24 (25.8%) |  |
| OSKIRA 3 (46) | 20 (43.5%) | 26 (56.5%) | 14 (30.4%) |  |
|  |  |  |  |  |
| C3M |  |  |  |  |
| All studies | 50% | 50% | 25% |  |
| OSKIRA 1 | 66 (52.8%) | 59 (47.2%) | 32 (25.65) | 0.0636  0.1920 |
| OSKIRA 2 | 51 (54.8%) | 42 (45.2%) | 21 (22.6%) |  |
| OSKIRA 3 | 16 (34.8%) | 30 (65.2%) | 13 (28.3%) |  |

## Sample size estimation using C1M for clinical trial enrichment (hypothesis generation)

In order to evaluate the potential use of C1M as a clinical trial tool for enrichment of progressors we calculated the sample sizes required for a statistical significance of 5%; power of 90% for relative treatment effects (reduction of in numbers of progressors) of 20%, 30% and 40% when compared to placebo, assuming progression rates of 10, 25 and 40% (table S3). If the progression rate is 10% the estimated number of patients needed would be of 8602, 3628 and 1930, if we expect treatment effects of 20, 30 and 40% respectively. In the current study, we saw that patients with low C1M had a progression rate of approx. 25% and those with a very high level had a progression rate of 40%. Thus, we calculated the estimated number of patients based on these numbers. If the progression rate for the population is 25%, the number of patients needed would be of 2928, 1248 and 670, if we expect relative treatment effects of 20, 30 and 40%, respectively. These patient numbers could be reduced to 1510, 652 and 354 (table S3) respectively. This corresponds to approx. a 50% reduction in number of patients needed in a radiographic progression clinical trial the OSKIRA studies.

### Table S3. Sample size estimations

| Progression rate | 20% reduction  N (%) | 30% reduction  N (%) | 40% reduction  N (%) |
| --- | --- | --- | --- |
| 10% | 8602 (100%) | 3628 (100%) | 1930 (100%) |
| 25% | 2928 (44%) | 1248 (44%) | 670 (45%) |
| 40% | 1510 (18%) | 652 (18%) | 354 (18%) |
